# Indole Signaling at the Host-Microbiota-Pathogen Interface

**DOI:** 10.1128/mBio.01031-19

**Published:** 2019-06-04

**Authors:** Aman Kumar, Vanessa Sperandio

**Affiliations:** a Department of Microbiology, University of Texas Southwestern Medical Center, Dallas, Texas, USA; b Department of Biochemistry, University of Texas Southwestern Medical Center, Dallas, Texas, USA; Johns Hopkins Bloomberg School of Public Health

**Keywords:** *Citrobacter rodentium*, CpxA, indole, enterohemorrhagic *E. coli* (EHEC), locus of enterocyte effacement (LEE), microbiota

## Abstract

Microbial establishment within the gastrointestinal (GI) tract requires surveillance of the gut biogeography. The gut microbiota coordinates behaviors by sensing host- or microbiota-derived signals. Here we show for the first time that microbiota-derived indole is highly prevalent in the lumen compared to the intestinal tissue. This difference in indole concentration plays a key role in modulating virulence gene expression of the enteric pathogens enterohemorrhagic Escherichia coli (EHEC) and Citrobacter rodentium. Indole decreases expression of genes within the locus of enterocyte effacement (LEE) pathogenicity island, which is essential for these pathogens to form attaching and effacing (AE) lesions on enterocytes. We synthetically altered the concentration of indole in the GI tracts of mice by employing mice treated with antibiotics to deplete the microbiota and reconstituted with indole-producing commensal Bacteroides thetaiotaomicron (*B. theta*) or a *B. theta* Δ*tnaA* mutant (does not produce indole) or by engineering an indole-producing C. rodentium strain. This allowed us to assess the role of self-produced versus microbiota-produced indole, and the results show that decreased indole concentrations promote bacterial pathogenesis, while increased levels of indole decrease bacterial virulence gene expression. Moreover, we identified the bacterial membrane-bound histidine sensor kinase (HK) CpxA as an indole sensor. Enteric pathogens sense a gradient of indole concentrations in the gut to probe different niches and successfully establish an infection.

## Abstract (precis)

Pathogens sense and respond to several small molecules within the GI tract to modulate expression of their virulence repertoire. Indole is a signaling molecule produced by the gut microbiota. Here we show that indole concentrations are higher in the lumen, where the microbiota is present, than in the intestinal tissue. The enteric pathogens EHEC and C. rodentium sense indole to downregulate expression of their virulence genes, as a read-out of the luminal compartment. We also identified the bacterial membrane-bound HK CpxA as an indole sensor. This regulation ensures that EHEC and C. rodentium express their virulence genes only at the epithelial lining, which is the niche they colonize.

## INTRODUCTION

There is a plethora of signals present in the human gut that mediate host-microbiota communication to maintain a homeostatic gastrointestinal (GI) environment (1, 2). The colon contains tryptophan derivatives such as indole, which is a microbiota-derived signaling molecule (3). Indole is also known to be absorbed by host cells and helps strengthen the integrity of the intestinal barrier and is regarded as a beneficial chemical cue within microbe-host interactions (4). Indole is synthesized by tryptophanase, the enzyme that catalyzes L-tryptophan conversion to indole (3) and is encoded by the *tnaA* gene. Both Escherichia coli (the predominant component of the *Gammaproteobacteria* phylum in the intestine) and Bacteroides thetaiotaomicron (*B. theta*) (one of the prominent species in the *Bacteroidetes* phylum [5]) have a *tnaA* gene (BT_1492 is the *B. theta* homolog [5]) and produce indole. The concentration of indole present in the human colon is not known. However, commensal and pathogenic strains of E. coli have been shown to produce approximately 500 μM indole in cultures (4), and the concentration of indole in human stools has been detected to be between 250 and 1,000 μM (6, 7). The continuous production of indole by the microbiota in the lumen and its absorption by the host cells suggest that a gradient concentration of indole exists in the intestine.

**FIG 1:**
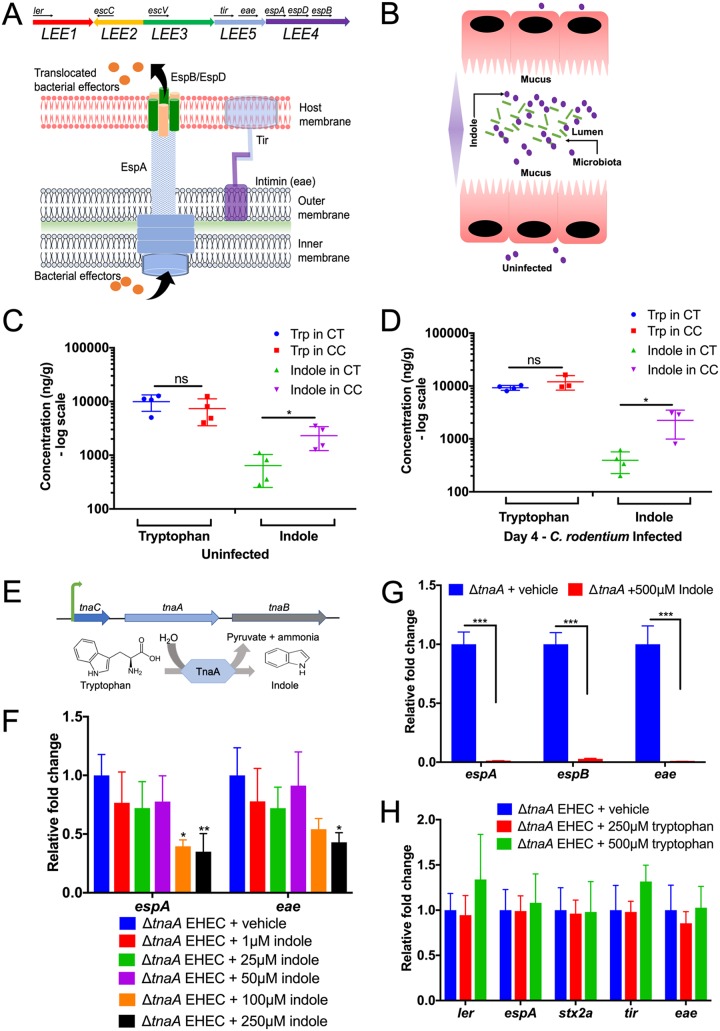
An indole concentration gradient is important to regulate virulence gene expression. (A, top) Schematic of the LEE pathogenicity island (*LEE1* to *LEE5* operons) and the representative virulence genes used in qRT-PCR experiments. (Bottom) Cartoon depicts the type III secretion system (encoded by the LEE) used by EHEC to inject bacterial effectors into the host cell. (B) Schematic representation for the indole concentration gradient in the human gut. Indole is produced by the microbiota in the lumen, where its concentration is higher. It is absorbed by epithelial cells, with its concentration decreasing at the epithelial lining. (C and D) Mass spectrometry measurement of indole and tryptophan (Trp) concentrations from colon tissues (CT) and colon content (CC) of C3H/HeJ mice before infection (C) and day 4 postinfection (D). Two-tailed unpaired *t* test was used to perform statistical analysis (*n* = 3 or 4 mice per group). Error bars represent standard errors of means (SEM). Values that are significantly different (*P* < 0.05) are indicated by a bar and asterisk. Values that are not significantly different (ns) are indicated. (E) Scheme of the EHEC *tna* operon depicting indole production resulting from tryptophan metabolism. (F) qRT-PCR for the expression of select virulence genes under increasing indole concentrations ranging from 1 to 250 μM. *P* values were determined by using one-way ANOVA followed by Bonferroni’s multiple-comparison test. Error bars represent standard deviations (SD). *, *P* < 0.05; **, *P* < 0.01. Experiments were performed in anaerobic conditions using low-glucose DMEM, and samples were harvested in late log phase. Data are representative of at least three independent experiments with three biological replicates and three technical replicates. Fold change were calculated relative to *rpoA* as an internal control. See also Fig. S1 in the supplemental material. (G) qRT-PCR for the expression of select virulence genes grown with 500 μM indole. Statistics were performed using unpaired *t* test followed by multiple comparison by Bonferroni-Dunn method. ***, *P* < 0.001. Experiments were performed under anaerobic conditions using low-glucose DMEM, and samples were harvested in late log phase. Data are representative of at least three independent experiments with three biological replicates and three technical replicates. Fold change were calculated relative to *rpoA* as an internal control. See also Fig. S1. (H) qRT-PCR analysis for the expression of virulence genes with various tryptophan concentrations. Statistics were calculated using ANOVA followed by Bonferroni’s multiple-comparison test. (F, G, and H) Experiments were performed in anaerobic conditions using low-glucose DMEM, and samples were harvested in late log phase. Data are representative of at least three independent experiments with three biological replicates and three technical replicates. Fold change were calculated relative to *rpoA* as an internal control. See also Fig. S1.

Enteric pathogens exploit intestinal chemistry to program virulence gene expression, leading to successful colonization of the GI tract (1). EHEC colonizes the human colon leading to outbreaks of bloody diarrhea and hemolytic-uremic syndrome (HUS) worldwide (8). EHEC virulence determinants include the production of the potent Shiga toxin that causes HUS and the genes necessary for the attaching and effacing (AE) lesion formation on enterocytes. AE lesion formation requires genes contained within the locus of enterocyte effacement (LEE) pathogenicity island (PI) (9). The LEE region contains five major operons, *LEE1* to *LEE5* (10) (Fig. 1A), which encode a type III secretion system (T3SS) (11), an adhesin (intimin) (12) and its receptor (Tir) (13), and effector proteins (14). T3SSs are molecular syringes that translocate bacterial effectors into the host cells, leading to changes in signaling and actin remodeling, culminating in the formation of AE lesions and contributing to overall EHEC pathogenesis (15). The *ler* gene (within the *LEE1* operon) encodes the master regulator of the LEE genes (10) (Fig. 1A). Transcription of *ler* is regulated by multiple bacterium- and host-derived signals (16). This exquisite regulation is necessary because deregulation of the LEE-encoded T3SS in EHEC constitutes an energy burden that hampers this pathogen’s ability to successfully compete with the enteric microbiota for a colonization niche (17, 18).

10.1128/mBio.01031-19.3 FIG S1 High expression of virulence genes is observed in late logarithmic phase. Download FIG S1, PDF file, 0.3 MB. Copyright © 2019 Kumar and Sperandio. 2019 Kumar and Sperandio This content is distributed under the terms of the Creative Commons Attribution 4.0 International license.

In this study, we show that self- and microbiota-derived indole decreases EHEC and Citrobacter rodentium LEE gene expression *in vitro* and during murine infection. We map the indole signaling cascade through RNA-Seq and identify the histidine sensor kinase CpxA as an indole sensor. Moreover, we also show that a gradient of indole exists within the mammalian GI tract. Indole is more prevalent in the lumen, where it is synthesized by the microbiota, and is decreased at the epithelium surface, where it is absorbed by host cells.

## RESULTS

### Exogenous indole decreases EHEC virulence gene expression.

Indole is synthesized from the amino acid tryptophan by the bacterial TnaA enzyme. Its levels within the mammalian intestine are not known. It has been shown that E. coli growing *in vitro* produces 500 μM indole (4) and that the concentration of indole in human stools is estimated to be between 250 and 1,000 μM (6, 7). Indole is produced by the microbiota that reside in the lumen and is absorbed by intestinal epithelial cells, suggesting that higher indole concentrations would be present in the lumen (4) (Fig. 1B). Congruent with this hypothesis, the concentration of indole is significantly higher in the lumen of the colon than in colonic tissues, while tryptophan concentrations remain unchanged in these compartments during either uninfected or C. rodentium-infected conditions (Fig. 1C and D).

EHEC has a tryptophanase (*tnaA*) gene and can produce its own indole (Fig. 1E). To assess the role of the indole gradient in regulating the expression of virulence genes, a mutant of EHEC that lacks *tnaA* and cannot produce its own indole was constructed. Treatment of Δ*tnaA* EHEC with increasing concentration of indole decreases the expression of virulence genes (LEE genes *espA*, *espB*, and *eae*) (Fig. 1F and G; see also Fig. S1A and B in the supplemental material). Importantly, there is no effect of its precursor molecule tryptophan on the expression of virulence genes (Fig. 1H). These data emphasize that indole specifically acts as a signaling molecule.

### Endogenous and exogenous sources of indole decrease EHEC virulence gene expression.

**FIG 2:**
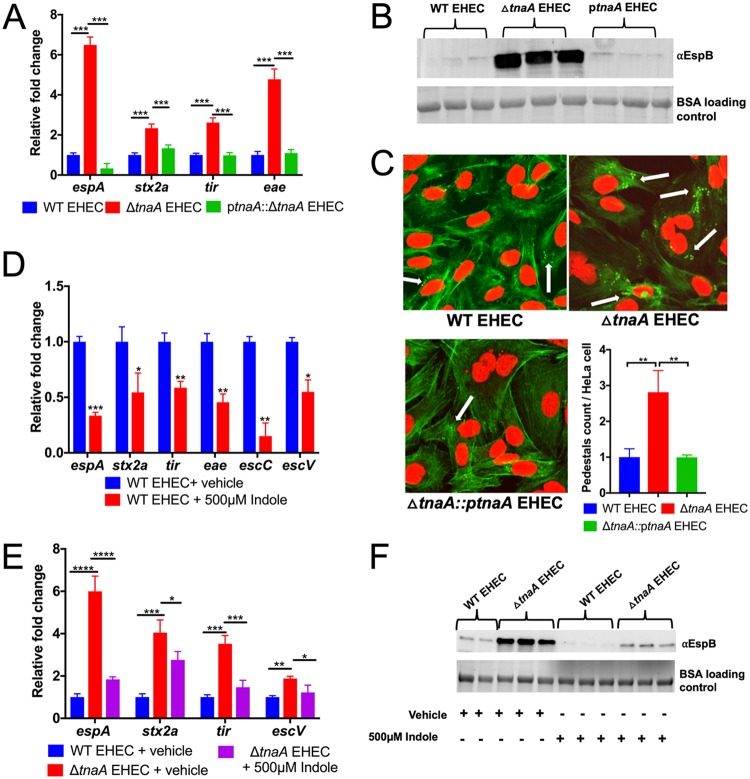
Indole decreases the expression of virulence genes. (A) qRT-PCR analysis to compare the expression of select virulence genes from WT EHEC, Δ*tnaA* EHEC, p*tna* (*tna* gene on a plasmid). Experiments were performed under anaerobic conditions using low-glucose DMEM, and samples were harvested in late log phase. Data are representative of at least three independent experiments with three biological replicates and three technical replicates. Fold change was calculated relative to *rpoA* as an internal control. Statistical analysis was performed using one-way ANOVA, followed by Bonferroni’s multiple-comparison test. ***, *P* < 0.001. (B) Western blot analysis of secreted protein EspB. EHEC (WT, Δ*tnaA*, or p*tna)* were grown in low-glucose DMEM under anaerobic conditions. Cells were harvested in late logarithmic phase at the same OD_600_ (same number of bacterial cells), and supernatants were concentrated for secreted proteins. BSA is used as a loading control to ensure no variability in the concentration step. αEspB, anti-EspB antibody. (C) Fluorescein actin staining analysis. HeLa cells were infected with WT EHEC, Δ*tnaA* EHEC, or p*tna* EHEC. At 5 h postinfection, cells were washed and stained with FITC-phalloidin to visualize actin (green) and propidium iodide to stain for bacteria and nuclei (red). Pedestals were visualized as green puncta (white arrows). Pedestals were enumerated for each field, with each field containing approximately 20 cells. The number of pedestals per infected cell was quantified (*n* = 3). Error bars represent standard deviations. **, *P* < 0.01 by one-way ANOVA. (D) qPCR for assessing the effect of addition of 500 μM indole on virulence gene expression of WT EHEC grown anaerobically. Statistical analysis was performed using unpaired *t* test, followed by multiple comparison by Bonferroni-Dunn method. *, *P* < 0.05; **, *P* < 0.01; ***, *P* < 0.001. (E) qRT-PCR expression analysis of select virulence genes from WT EHEC, Δ*tnaA* EHEC, and Δ*tnaA* EHEC with 500 μM exogenously added indole. Bacterial cells were grown under anaerobic conditions. Statistical significance was calculated using one-way ANOVA, followed by Bonferroni’s multiple-comparison test. *, *P* < 0.05; **, *P* < 0.01; ***, *P* < 0.001; ****, *P* < 0.0001. (F) Western blot comparison of the secreted protein EspB from WT and Δ*tnaA* EHEC grown anaerobically in the presence or absence of 500 μM indole. BSA was used as a loading control. All qRT-PCR data are representative of at least three independent experiments with three biological replicates and three technical replicates. Fold change was calculated relative to an internal control *rpoA*. Error bars represent standard deviations. See also Fig. S1 and S2.

Wild-type (WT) EHEC produces indole because it harbors *tnaA*. Congruent with indole decreasing LEE gene expression (Fig. 1F), LEE-encoded (*espA*, *tir*, and *eae*) as well as non-LEE-encoded Shiga toxin (*stx2a*) expression is increased in Δ*tnaA* EHEC, and this phenotype can be complemented (Fig. 2A and B). It is noteworthy that the increase in LEE (*espA* and *eae* genes) and *stx2a* expression in Δ*tnaA* is more pronounced in late logarithmic growth phase (Fig. S1C to E), which is when *tnaA* expression is increased (Fig. S1F). The LEE is required for AE lesion formation. AE lesions are characterized by remodeling of the epithelial cell cytoskeleton forming a pedestal-like structure that cups EHEC (8). In accordance with the LEE expression phenotype, AE lesion formation is enhanced in the Δ*tnaA* mutant strain compared to WT and complemented strains (Fig. 2C). Exogenous addition of 500 μM indole to both WT and Δ*tnaA* EHEC decreased the expression of virulence genes, as well as AE lesion formation. Although exogenous indole dampened virulence-related phenotypes, it had no effect on bacterial growth or viability (Fig. 1G and 2D and Fig. S2A to D). These findings are congruent with previous literature that reported that only high indole concentrations (5 mM), which are 15-fold higher than the physiological concentration measured in E. coli, have toxic effects on growth (19). More importantly, exogenously added indole (500 μM) to Δ*tnaA* EHEC rescues the WT EHEC phenotype (Fig. 2E and F). This combined with the fact that the Δ*tnaA* EHEC has no growth defect compared to WT EHEC (Fig. S1C) indicates that indole-dependent LEE regulation is not due to a metabolic defect caused by deletion of *tnaA*, but to indole signaling.

10.1128/mBio.01031-19.4 FIG S2 High indole concentration decreases EHEC virulence without affecting its growth. Download FIG S2, PDF file, 0.5 MB. Copyright © 2019 Kumar and Sperandio. 2019 Kumar and Sperandio This content is distributed under the terms of the Creative Commons Attribution 4.0 International license.

**FIG 3:**
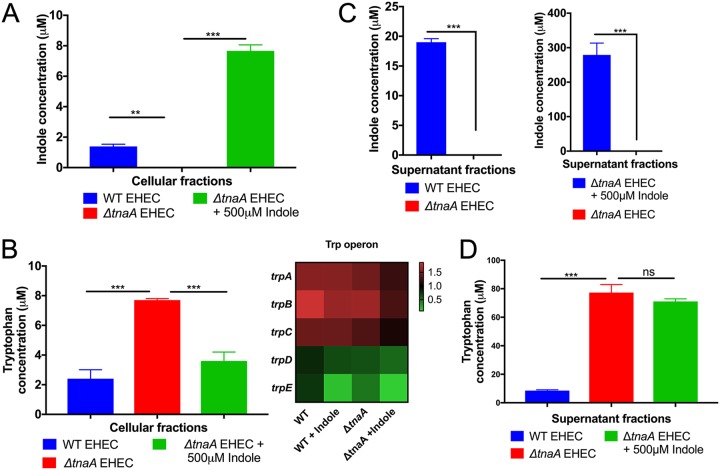
Intracellular and extracellular indole levels in EHEC. (A and B) Mass spectrometry measurement of indole and tryptophan obtained from the cellular fractions. WT EHEC, Δ*tnaA* EHEC, and Δ*tnaA* EHEC with 500 μM indole were grown anaerobically in low-glucose DMEM. After 5 h, cells were centrifuged to separate the cellular and supernatant fractions. (A) Addition of 500 μM indole to Δ*tnaA* EHEC rescued the WT levels of indole in the cellular fraction. (B) Tryptophan concentrations were measured as a control. The heat map to the right indicates the results of RNA sequencing analysis, revealing a decrease in the expression of tryptophan biosynthesis genes (*trp* operon) in the presence of indole. Statistics were performed using one-way ANOVA. (C) Mass spectrometry measurement of indole obtained from the supernatant fraction. Statistics were performed using unpaired *t* test. (D) Tryptophan concentration obtained from the supernatant fraction. Note that the concentration of tryptophan in DMEM is 78.431 μM. One-way ANOVA was performed to calculate statistical significance. Values that are significantly different are indicated by asterisks as follows: **, *P* < 0.01; ***, *P* < 0.001. *n* = 3 per group. Error bars indicate standard deviations (SD). See also Fig. S1.

Indole moonlights as a metabolite and a signaling molecule, and as an extracellular signal, it has been reported to require minimal concentrations of 500 μM to be active (3). We measured the indole and tryptophan concentrations within bacterial cells and in supernatants in our *in vitro* experiments. Exogenous addition of 500 μM indole to Δ*tnaA* EHEC led to the recovery of a small fraction of indole from the cellular fraction, which was comparable to the amount of indole present in the cellular fraction of WT EHEC (Fig. 3A). Indole has been reported to freely diffuse across membranes (20). Our data suggest that either a high concentration of exogenous indole is necessary to cross the bacterial membrane or that once indole enters the cell, it is quickly shuffled in as a metabolite, which could account for the decrease in its cellular concentration. WT EHEC can metabolize tryptophan; therefore, there is less tryptophan in the cellular fraction of WT EHEC than in Δ*tnaA* EHEC. However, less tryptophan was obtained from Δ*tnaA* EHEC treated with 500 μM indole, because the addition of indole decreases expression of the tryptophan synthetic pathway (Fig. 3B). Measurements of the indole concentrations from supernatants of WT or Δ*tnaA* EHEC in the presence or absence of 500 μM indole suggest that WT EHEC converts most of the tryptophan to indole, while there was no change in the concentrations of tryptophan in the Δ*tnaA* EHEC (Fig. 3C and D).

### CpxA is an indole sensor.

**FIG 4:**
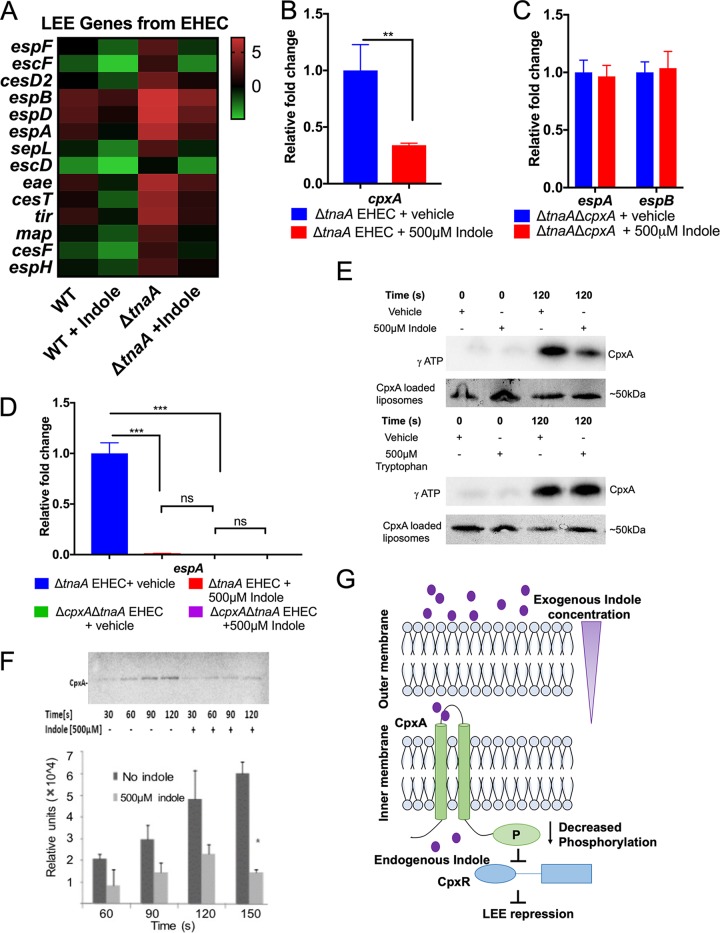
Indole regulates the virulence gene expression of EHEC through the histidine sensor kinase CpxA. (A) Heat map showing expression of LEE genes. WT EHEC and Δ*tnaA* EHEC in the presence or absence of 500 μM indole were grown until late logarithmic phase anaerobically. RNA sequencing was performed on the extracted RNA, and data analysis were conducted using ArrayStar. (B) qRT-PCR analysis on *cpxA* confirming that the presence of indole decreases its expression. △*tnaA* EHEC was grown anaerobically in the presence or absence of 500 μM indole. Statistical significance was calculated using unpaired *t* test. **, *P* < 0.05. (C) qRT-PCR showing that indole has no further effect on *LEE4* (*espA* and *espB*) transcription in the absence of the CpxA sensor under anaerobic growth conditions. (D) qRT-PCR analysis comparing the expression of *espA* in the absence of *cpxA* with or without indole during anaerobic growth. Statistical analysis was performed using one-way ANOVA. In panels B to D, Δ*tnaA* EHEC was used as a background strain to avoid interference of endogenous indole. Fold change was calculated relative to *rpoA* as an internal control. All data are representative of two independent experiments with three biological and three technical replicates. Error bars represent standard deviations (SD). Values that are significantly different are indicated by asterisks as follows: **, *P* < 0.01; ***, *P* < 0.001. Values that are not significantly different (ns) are indicated. (E) Autophosphorylation of CpxA (loaded in liposomes) in the presence or absence of 500 μM (each) indole and tryptophan. Samples were run on 12% stain-free SDS-PAGE gel. CpxA loaded liposomes were visualized using Chemi Doc. Autoradiographs were obtained by using a phosphorimager. Data are representative of three independent experiments. (F) Time course for the autophosphorylation of CpxA (loaded in liposomes) in the presence or absence of indole. Autophosphorylation signal was quantified using Image Quant. Results were compared by unpaired *t* test, followed by multiple comparison by Bonferroni-Dunn method (*n* = 3). Error bars indicate SD. *, *P* < 0.05. (G) Schematic representation for indole signaling. High indole concentrations allow indole to cross the outer membrane of the enteric pathogens EHEC and C. rodentium. The histidine kinase CpxA in the inner membrane senses the presence of indole and decrease its phosphorylation leading to its decreased downstream activity and subsequent LEE repression. See also Fig. S4 and S5.

To probe the indole transcriptome, RNA-Seq was performed comparing gene expression from WT or Δ*tnaA* EHEC in the presence or absence of indole (GEO accession no. GSE119440). As expected, expression of the LEE genes decreased with the addition of indole (Fig. 4A). Previous studies reported sensing of small-molecule signals by bacterial membrane-bound histidine sensor kinases (HKs) (17, 21). Upon sensing a signal, HKs can either increase or decrease their autophosphorylation states, which lead to phosphorylation or dephosphorylation of response regulators (RRs) that are mostly transcription factors. Phosphorylation of RRs increases their ability to bind to DNA and regulate gene expression, while dephosphorylation inhibits their function. The cognate HK and RR pairs constitute a two-component signaling system (TCS) (22). Additionally, the signals tend to regulate expression of their sensor HKs (21). Among all the TCSs, the CpxA-CpxR system was the most significantly regulated by indole (Fig. S3A and B and Fig. S4A to C). The expression of *cpxA* was confirmed to be decreased with the addition of indole (Fig. 4B and Fig. S4A and B). The Δ*tnaA* Δ*cpxA* EHEC does not respond to indole to decrease transcription of the LEE (*espA* and *espB*), *cpxR*, and *stx2a* genes (Fig. 4C and D and Fig. S5A and B). Also, the Δ*cpxA* EHEC has decreased LEE expression and does not respond to indole (Fig. S5C and D). Additionally, autophosphorylation of CpxA reconstituted in liposomes is decreased in the presence of indole, and this phenotype is not responsive to tryptophan (Fig. 4E and F). CpxA is necessary for LEE gene activation in EHEC and virulence in C. rodentium (23, 24) (a murine pathogen extensively used as an infection model for EHEC [25], given that EHEC is strictly a human pathogen and cannot colonize mice). Therefore, CpxA senses indole as a signaling molecule to dephosphorylate itself, which can lead to dephosphorylation of CpxR, decreasing LEE gene expression (Fig. 4G). The CpxAR system is known to be activated by envelope perturbations; hence, at high toxic indole levels (2 mM), an E. coli *cpxR* mutant is responsive to indole, because of perturbations of membrane integrity (26).

10.1128/mBio.01031-19.5 FIG S3 CpxA identified as an indole sensor. Download FIG S3, PDF file, 0.5 MB. Copyright © 2019 Kumar and Sperandio. 2019 Kumar and Sperandio This content is distributed under the terms of the Creative Commons Attribution 4.0 International license.

10.1128/mBio.01031-19.6 FIG S4 Indole signaling occurs through CpxA-CpxR node. Download FIG S4, PDF file, 0.6 MB. Copyright © 2019 Kumar and Sperandio. 2019 Kumar and Sperandio This content is distributed under the terms of the Creative Commons Attribution 4.0 International license.

10.1128/mBio.01031-19.7 FIG S5 A *cpxA* mutant is indole irresponsive and is attenuated for pathogenesis. Download FIG S5, PDF file, 0.2 MB. Copyright © 2019 Kumar and Sperandio. 2019 Kumar and Sperandio This content is distributed under the terms of the Creative Commons Attribution 4.0 International license.

### Self-produced indole decreases C. rodentium virulence in mice.

**FIG 5:**
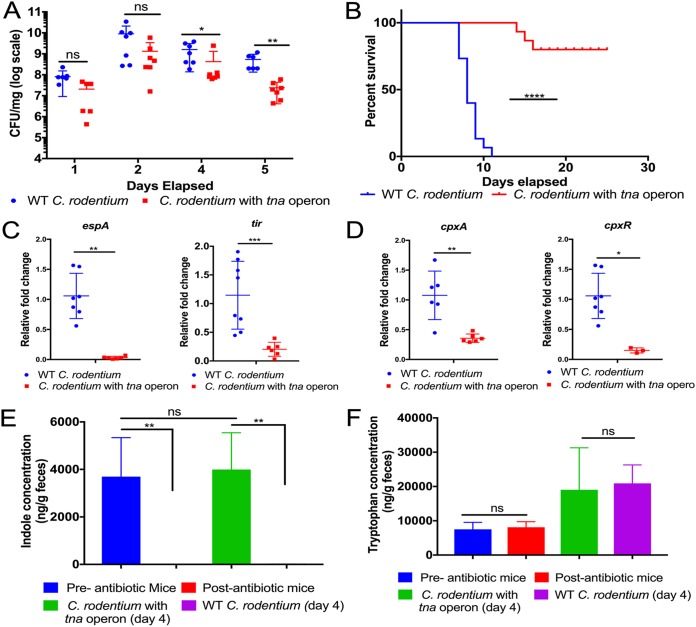
Indole limits C. rodentium colonization and pathogenesis in mice. (A) Colonization of C3H/HeJ mice with either WT C. rodentium or C. rodentium with the *tna* operon in microbiota-depleted mice. *P* value was determined by using Mann-Whitney *U* test. Each symbol represents the value for an individual mouse. Error bars indicate standard errors of means (SEM). (B) Survival analysis of mice infected with either WT C. rodentium or C. rodentium with the *tna* operon. A total of *n* = 15 mice per group were used for the study. Statistical significance was calculated using log rank (Mantel-Cox) test. ****, *P* < 0.0001. (C and D) Expression of bacterial virulence genes during murine infection. Stools from mice infected with either WT C. rodentium or C. rodentium with *tna* operon were collected, and RNA was extracted. qRT-PCR analysis was performed on select virulence genes *espA* and *tir* (C) and the indole sensor *cpxA* and its cognate response regulator *cpxR* (D). Fold change was determined relative to an internal control, *rpoA*. Each symbol represents the value for an individual mouse. Statistical significance was calculated using Mann Whitney *U* test. Error bars represent standard errors of means (SEM). Values that are significantly different are indicated by asterisks as follows: *, *P* < 0.05; **, *P* < 0.01; ***, *P* < 0.001. (E and F) Measurement of indole (E) or tryptophan (F) concentrations from fecal samples of mice infected with either WT C. rodentium or C. rodentium with the *tna* operon using mass spectrometry. *N* = 4 per group was used. Statistical analysis was performed using one-way ANOVA, followed by Bonferroni’s multiple-comparison test. Error bars indicate standard errors of means (SEM). **, *P* < 0.01; ns, not significant. See also Fig. S4, S5, and S6.

Although C. rodentium is extensively used as a surrogate murine model for EHEC infections (27), it lacks the TnaA enzyme and cannot produce its own indole. To assess the effect of indole *in vivo*, the EHEC *tna* operon was inserted within the *lacZ* locus (*lacZ* does not contribute to C. rodentium murine infection [28]) of C. rodentium, which now produces indole (Fig. S6A and B). The murine microbiota was depleted as previously described (29, 30) to exclusively investigate the effect of self-produced indole in C. rodentium pathogenesis. Indole decreased the overall bacterial burden (Fig. 5A), mortality (Fig. 5B), and virulence gene expression (LEE genes *espA* and *tir*) (Fig. 5C). The levels of indole and tryptophan *in vivo* were measured by using mass spectrometry (Fig. 5E and F). This establishes that indole decreases the expression of virulence genes, as well as C. rodentium virulence in mice. However, indole-producing C. rodentium colonizes and causes mortality similar to that caused by WT C. rodentium in a microbiota-intact mouse (Fig. S6C and D). This is because similar amounts of indole were recovered from the stools of these mice, due to the high levels of indole being produced by the complex microbiota (Fig. S6E). These findings suggested that microbiota-derived or self-produced indole had similar effects in dictating C. rodentium infectivity. Indole signaling occurs through the CpxA HK (Fig. 4B to G and Fig. S5A to D). However, a C. rodentium *cpxRA* deletion strain is heavily attenuated and cannot colonize mice (24), as is a Δ*cpxA* strain (Fig. S5E and F), thereby limiting the ability to analyze indole signaling through the CpxRA system *in vivo*. Importantly, expression of *cpxA* and *cpxR* is decreased during murine infection with C. rodentium containing the *tna* operon (Fig. 5D). Hence, when enteric pathogens encounter high indole concentrations in the gut, indole is sensed by CpxA, which causes decreased expression of the LEE by modulating the expression and activity of CpxA (Fig. 4G).

10.1128/mBio.01031-19.8 FIG S6 Self-produced indole or microbiota-derived indole dictates C. rodentium infectivity in a similar fashion. Download FIG S6, PDF file, 0.1 MB. Copyright © 2019 Kumar and Sperandio. 2019 Kumar and Sperandio This content is distributed under the terms of the Creative Commons Attribution 4.0 International license.

### Microbiota-produced indole decreases C. rodentium virulence in mice.

**FIG 6:**
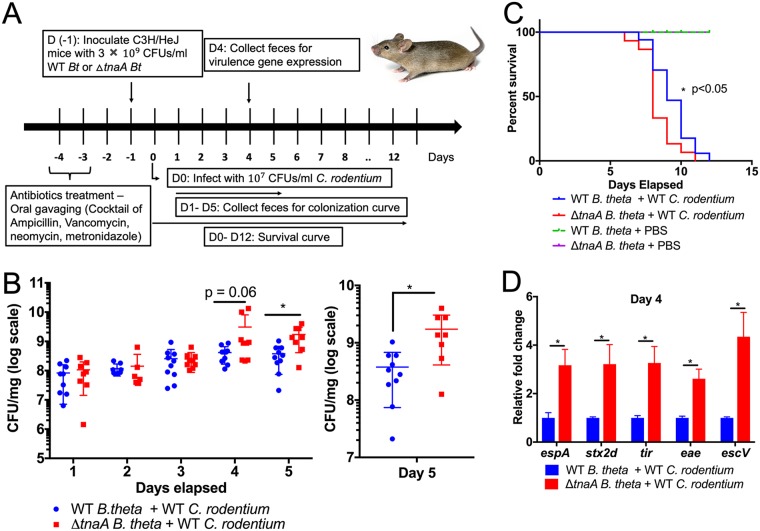
Synthetically altering the microbiota to remodel gut indole concentration dictates C. rodentium infectivity. (A) Schematic representation of the microbiota remodeling and C. rodentium murine infection experiments. D, day; *Bt*, Bacteroides thetaiotaomicron. (B to D) C3H/HeJ mice precolonized with either WT B. thetaiotaomicron (*B. theta*) or Δ*tnaA B. theta* were infected with WT C. rodentium. (B) Mice were monitored for the C. rodentium colonization. Statistical significance was calculated using two-sided Mann-Whitney *U* test. Each symbol indicates the value for an individual mouse. (C) Survival of mice. *P* value was determined by using log rank (Mantel-Cox) test. A total of *n* = 15 mice per group was used. (D) Expression of select virulence genes. Statistical significance was calculated using unpaired *t* test, followed by multiple comparison using Bonferroni-Dunn method. Error bars represent standard errors of means (SEM). *, *P* < 0.05. See also Fig. S7.

**FIG 7:**
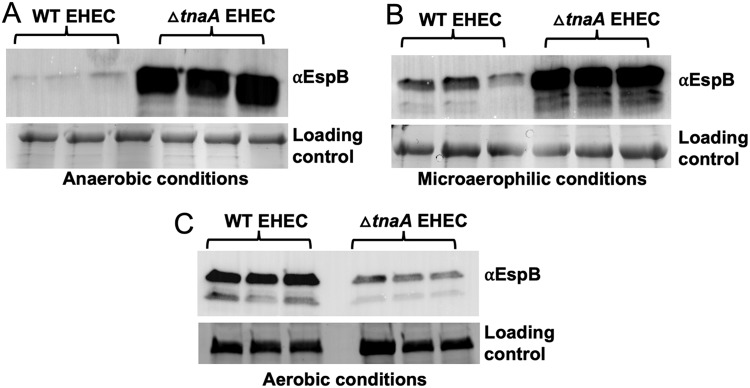
EHEC changes its virulence expression in different oxygenation conditions. (A to C) WT and △*tnaA* EHEC were grown under anaerobic (A), microaerophilic (B), or aerophilic (C) conditions. The cells were harvested in late log phase. The supernatants were collected, and the secreted protein fractions were probed using EspB antibody. BSA was used as a loading control. See also Fig. S1.

Analysis of more than 1,000 metagenomes of American, European, and Chinese subjects showed that the tryptophanase genes are highly abundant and are present in one out of six microbial species (31). Within the human gut microbiota, tryptophanases are more prominent in *Bacteroides* species (31). Therefore, *Bacteroides* species occupying the anaerobic lumen are mainly responsible for production of the majority of the indole in the gut. To investigate the effect of microbiota-produced indole in C. rodentium murine infection, a *Bacteroides thetaiotamicron* (*B. theta*) Δ*tnaA* strain that cannot produce indole was constructed (Fig. S7A). The murine microbiota was depleted, and these animals were colonized with either WT or Δ*tnaA B. theta* (Fig. 6A). Microbiota-depleted and Δ*tnaA B. theta*-colonized mice had no indole detected in their stools, while mice colonized with WT *B. theta* had abundant levels of indole (Fig. S7B). Of note, both WT and Δ*tnaA B. theta* colonized mice to similar levels (Fig. S7C). The presence of indole-producing *B. theta* led to decreased C. rodentium burden and pathogenesis with prolonged survival of mice (Fig. 6B and C). C. rodentium LEE (*espA*, *tir*, *eae*, and *escV*) and *stx2a* gene expression was increased in mice colonized with Δ*tnaA B. theta* compared to mice colonized with the indole-producing WT *B. theta* (Fig. 6D). Hence, microbiota-produced indole in the lumen limits virulence of enteric pathogens. The luminal environment is anaerobic, while the epithelial surface is microaerophilic and becomes aerophilic during C. rodentium infection, leading to pathogen expansion because it is aerotolerant (32). Congruent with this scenario, EHEC LEE expression is increased during aerophilic conditions and decreases when oxygen tension is diminished, being barely detectable under anaerobiosis. Importantly, this decrease under anaerobiosis is dependent on indole, given that a Δ*tnaA* EHEC expresses high levels of the LEE proteins under anaerobiosis (Fig. 7A to C).

10.1128/mBio.01031-19.9 FIG S7 Bacteroides thetaiotaomicron (*B. theta*) murine colonization is not affected by *tnaA.* Download FIG S7, PDF file, 0.2 MB. Copyright © 2019 Kumar and Sperandio. 2019 Kumar and Sperandio This content is distributed under the terms of the Creative Commons Attribution 4.0 International license.

## DISCUSSION

Enteric pathogens navigate the complex intestinal chemistry to find a suitable colonization niche. The microbiota plays an important part in shaping this chemistry (30, 33, 34) and has key interactions with enteric pathogens. These interactions are complex, with certain signals such as fucose decreasing EHEC LEE expression (17), while others such as succinate leads to increased pathogenesis (30). Both these signals are either made available or produced by a prominent member of the microbiota, *B. theta*. EHEC has a very low infectious dose, which is estimated to be 50 CFU (8), and colonizes the colon where the microbiota is very dense. In the colon, the major source for carbon is the mucus, which is decorated with sugars such as fucose. These sugars can be harvested by saccharolytic members of the microbiota, such as *B. theta*, and made available to other bacterial species that lack this capability (35). EHEC senses the fucose released from the mucus by *B. theta* to sense the lumen environment through the two-component system FusKR. In response to fucose, FusKR modulates EHEC’s metabolism to optimize its growth and decrease competition for carbon sources with commensal E. coli. It also decreases expression of the LEE genes that are not necessary in this gut compartment (17). As infection progresses, mucinases of EHEC and C. rodentium degrade the mucus layer (30). Importantly, because the main source of sugars in the colon is the mucus, this pathogen-induced obliteration of the mucus layer creates a nutrient-poor environment, which is gluconeogenic. Additionally, in this scenario, colonization of mice by *B. theta* profoundly changes the metabolic landscape of the murine gut, with organic acids such as succinate being enhanced (30, 36, 37). Succinate is then sensed by Cra to induce LEE expression at the epithelial lining (30, 38).

Indole is known to be produced by the microbiota and is present in the intestine. Indole has also been shown to be absorbed by the intestinal epithelial cells to improve barrier function (4). However, whether concentrations of indole vary between the lumen versus the vicinity of the epithelium was not established. Here we show that indole concentrations are indeed higher in the lumen compared to the epithelium (Fig. 1B to D). These data suggest that indole can also be used as a signal sensed by enteric pathogens to navigate the gut biogeography. Here we show that by sensing indole concentrations in the gut, EHEC and C. rodentium discriminate between the luminal environment, where expression of the T3SS is an unnecessary energy burden, from the epithelial surface, where T3SS expression is needed for host colonization. These findings establish a mechanism through which the microbiota confers resistance to pathogens. Moreover, they open the possibility that manipulation of indole concentrations in the GI tract by pre- or probiotics that produce indole can limit virulence of enteric pathogens.

## MATERIALS AND METHODS

### Strains, plasmids, and growth and culture conditions.

Strains and plasmids used in this study are listed in Table S1 in the supplemental material. WT enterohemorrhagic E. coli (EHEC) O157:H7 strain 86-24, Citrobacter rodentium (DBS770), and their isogenic mutants were routinely grown in LB overnight at 37°C. Bacteroides thetaiotaomicron (*B. theta*) VPI-5482 was routinely grown anaerobically overnight at 37°C in TYG medium. To express the type III secretion system (T3SS), low-glucose (1 g/liter) Dulbecco’s modified Eagle medium (DMEM) was used, as these conditions have been shown to induce the T3SS (38). Anaerobic growth was performed using either GasPak EZ anaerobe container system (Becton Dickinson) or Bactron EZ anaerobic chamber (Sheldon Manufacturing). For microaerophilic conditions, cultures were left standing in a 37°C incubator. For aerophilic conditions, cultures were grown under shaking conditions at 37°C and 250 rpm. Bacterial strains were grown in low-glucose DMEM, and the cultures were harvested in late logarithmic growth phase unless stated otherwise. HeLa cells were routinely cultured in high-glucose DMEM, defined as 4.5 g/liter glucose, DMEM, 10% FBS, and penicillin plus streptomycin plus glutamine (PSG) cocktail.

10.1128/mBio.01031-19.1 TABLE S1 Bacterial strains. Download Table S1, PDF file, 0.3 MB. Copyright © 2019 Kumar and Sperandio. 2019 Kumar and Sperandio This content is distributed under the terms of the Creative Commons Attribution 4.0 International license.

### Recombinant DNA techniques.

All primers for used for mutant and plasmid construction can be found in Table S2.

10.1128/mBio.01031-19.2 TABLE S2 Oligonucleotides used in this study. Download Table S2, PDF file, 0.1 MB. Copyright © 2019 Kumar and Sperandio. 2019 Kumar and Sperandio This content is distributed under the terms of the Creative Commons Attribution 4.0 International license.

### Construction of deletion mutants of EHEC and Citrobacter rodentium.

Isogenic mutants of EHEC 86-24 strain were created using the λ red recombination technique (39). Briefly, pKD4 was used to create the deletion PCR products. pKD46-expressing strain was used to perform the recombination and pCP20 to resolve the insertions. All mutant strains were confirmed by sequencing.

### Construction of deletion mutant of *B. theta*.

The *tnaA* mutant was created in the B. thetaiotaomicron VPI-5482 Δ*tdk* background, a gene encoding thymidine kinase as previously described (40). Briefly, the *tnaA* (BT1492) gene with ∼750-bp upstream and downstream sequence were ligated into a suicide vector, pExchange-tdk. The resulting construct was transformed into competent S17-1 λ *pir.* The knockout construct was conjugated in a *B. theta* Δ*tdk* strain by growing them together. The integrant were selected on BHI-blood agar plates with 200 μg/ml gentamicin and 25 μg/ml erythromycin. The integrants were cultured in TYG medium overnight and then plated onto BHI-blood agar plates containing 200 μg/ml 5-fluoro-2-deoxyuridine (FUdR) to select for successful recombinants. The Δ*tnaA B. theta* deletions were screened by PCR and confirmed by sequencing.

### Western blotting for secreted proteins.

From cultures grown in DMEM, secreted proteins were isolated as previously described (11). Ten micrograms of bovine serum albumin (BSA) was added to secreted protein samples as a loading control. Proteins were separated on a 5 to 20% SDS-polyacrylamide gel, transferred to a polyvinylidene fluoride (PVDF) membrane, and blocked with 3% milk in PBS containing 0.05% Tween (PBST). Membranes were probed with either anti-EspB or anti-EspA primary antibody, washed, and then incubated with a secondary rabbit antibody conjugated to streptavidin-horseradish peroxidase. Enhanced chemiluminescence (ECL) reagent was added, and the membranes were developed using the ChemiDoc touch imaging system (software 1.0.0.15) with Image Lab 5.2.1 software for image display.

### RNA extraction and qRT-PCR.

Strains were grown in the presence or absence of indole until late log phase. RNA from three biological replicates was extracted using the RiboPure bacterial isolation kit according to the manufacturer’s protocols (Ambion). Quantitative reverse transcription-PCR (qRT-PCR) was performed as follows. Briefly, 2 μg of diluted extracted RNA was converted to cDNA with addition of superscript, random primers, DTT, and dNTPs. Validated primers (Table S2) and SYBR green were added to the cDNA, and the mix was run in Quantstudio 6 flex (Applied Biosystems). Data were collected using QuantStudio real-time PCR software v1.3, normalized to endogenous *rpoA* levels, and analyzed using the comparative cycle threshold (*C_T_*) method. For all the *in vitro* experiments, error bars indicate standard deviations (SD). A *P* value of less than 0.05 was considered significant.

### Fluorescein actin staining assays.

Fluorescein actin staining (FAS) assays were performed as described previously (41). Briefly, HeLa cells were grown overnight to about 80% confluence at 37°C and 5% CO_2_ on coverslips in wells containing high-glucose (4.5 g/liter) DMEM. Prior to infection, fresh DMEM lacking antibiotics replaced overnight medium. To infect HeLa cells, late-log-phase bacterial cultures with equal CFU grown in low-glucose DMEM anaerobically for 5 h were used. HeLa cells were infected with an MOI of 100. After 5 h, the coverslips were washed, fixed, and permeabilized. The samples were treated with fluorescein isothiocyanate (FITC)-labeled phalloidin to visualize actin accumulation and propidium iodide to visualize bacterial DNA and HeLa nuclei. The coverslips were then mounted on slides and imaged with a confocal microscope (Zeiss LSM 880 confocal/multiphoton) at 60× resolution. The number of pedestals per HeLa cell was quantified. Replicate coverslips from multiple experiments were quantified, and statistical analyses were performed using Student’s unpaired *t* test, followed by multiple comparison by Bonferroni-Dunn method wherever required. A *P* value of less than 0.05 was considered significant.

### RNA sequencing library preparation and analysis.

Briefly, RNAs extracted from three biological replicates were used to perform RNA sequencing experiments. Sequencing was run at the University of Texas (UT) Southwestern Medical Center Next Generation Sequencing (NGS) core. RNA libraries were prepared using Illumina ScriptSeq Complete kit (Bacteria). RNA libraries were run on an Illumina HiSeq 2500 sequencer with SE-50. To analyze the data, DNASTAR Lasergene software was used. Reads were mapped to the Escherichia coli O157:H7 strain sakai genome (NCBI txid 386585). Data were analyzed using ArrayStar, and all experiments were normalized by reads assigned per kilobase of target per million mapped reads (RPKM). Statistical significance was calculated using Student’s *t* test followed by FDR (Benjamini Hochberg) correction. A *P* value of less than 0.05 was considered significant. All experiments were normalized by reads assigned per kilobase of target per million mapped reads (RPKM).

### Membrane protein purification.

E. coli (BL21 DE3) bacteria harboring pET21a-CpxA (His tagged) were grown until stationary phase (optical density [OD] of ∼0.7 to 0.8). Cultures were induced with 0.1 mM IPTG and grown further at 18°C overnight. The bacterial cultures were spun down at 10,000 rpm and 4°C for 30 min, and the pellets were stored at −80°C until further use. The bacterial pellets were resuspended in lysis buffer (50 mM Tris [pH 8.0], 150 mM NaCl, 2% lauryldimethylamine *N*-oxide [LDAO], 10% glycerol), and protease inhibitor cocktail (PIC) was added. The lysates were sonicated for 30 s (on) and 30 s off for 8 min at 85% amplitude, followed by centrifugation at 10,000 rpm and 4°C for 30 min. The supernatants were filtered using 0.22-μm filter tube. Ni-NTA beads (500 μl) were added to 40 ml of lysate and kept on a shaker for up to 2 h at 4°C to allow protein binding to the Ni beads. The bound lysates were passed through 5-ml disposable columns twice. The column was washed with 5 column volumes (CV) of wash buffer (50 mM Tris [pH 8.0], 150 mM NaCl, 30 mM imidazole). Proteins were eluted from the column by adding elution buffer (50 mM Tris [pH 8.0], 150 mM NaCl, 250 mM imidazole). The eluted proteins were concentrated using Amicon Ultra 15 centrifugal filter units (10 kDa), and excess imidazole was removed by adding 50 mM Tris (pH 8.0) and 150 mM NaCl as a dialyzing buffer. The protein concentration was estimated using Bradford assay and were immediately loaded on liposomes for autophosphorylation assays.

### Autophosphorylation assays.

The autophosphorylation experiments were performed as previously described (21). Liposomes were reconstituted as described previously (21). Briefly, 50 mg of E. coli phospholipids (Avanti Polar Lipids; 20 mg/ml in chloroform) were evaporated and then dissolved into 5 ml phosphate buffer containing 80 mg *N*-octyl-β-D-glucopyranoside. The solution was dialyzed overnight against phosphate buffer. The resulting liposome suspension underwent freeze/thawing in liquid N_2_. The liposomes were stored at −80°C until further use. The liposomes (1 ml) were then destabilized by the addition of 5.8 mg dodecylmaltoside, and CpxA-His was added in the ratio of 40:1, stirring at room temperature for 10 min. Biobeads (58 mg) were then added to remove the detergent, and the resulting solution was allowed to incubate at 4°C overnight. The supernatant was then incubated with fresh Biobeads (58 mg) for another hour. The resulting liposomes containing reconstituted CpxA-His was used for autophosphorylation experiments. Ten microliters of the CpxA-His liposome was adjusted to 10 mM MgCl_2_,1 mM DTT, 500 μM indole, 500 μM tryptophan or no signal, freeze/thawed rapidly in liquid N_2_, and kept at room temperature for 1 h. [γ-^32^P]ATP (0.25 μl) was added to each reaction mixture. At each time point, 2 μl of 5× SDS loading buffer was added to stop the reaction. The samples were run on 12% SDS-PAGE according to standard procedures and visualized via a phosphorimager (Typhoon FLA 9500; GE).

### Indole colorimetric test.

Portions (50 μl) of bacterial cultures were added with 50 μl of either *p*-dimethylaminocinnamaldehyde (DMACA) indole reagent droppers (Becton Dickinson) or with BBL indole reagent droppers (Becton Dickinson). The presence of indole was detected by change in color. The presence of indole was observed by formation of green coloration on treatment with DMACA indole reagent and pink color with BBL indole reagent dropper.

### Murine infections.

Three- to 4-week-old female C3H/HeJ mice were purchased from The Jackson Laboratory and housed in a specific-pathogen-free facility at the UT Southwestern Medical Center. All experiments were performed under IACUC-approved protocols.

### Microbiota depletion method.

At 3 to 4 weeks of age, female C3H/HeJ mice were orally administered a combination of four antibiotics, ampicillin, neomycin, metronidazole, and vancomycin (Sigma-Aldrich), via oral gavage for 2 days to deplete gut microbiota (5 mg of each antibiotic per mouse per day). Fecal pellets were collected before and after antibiotic treatment to confirm depletion of the gut microbiota. Feces were resuspended in PBS at 1 g/ml and plated on brain heart infusion (BHI)-blood agar plates containing no antibiotics. Colony counts were performed after 48-h incubation at 37°C under both aerobic and anaerobic conditions.

### *B. theta* reconstitution experiment.

Following antibiotic treatment, half of the mice were administered 3 × 10^9^ CFU WT *B. theta*, while the remainder of the mice were given 3 × 10^9^ CFU Δ*tnaA B. theta* via oral gavage. Twenty-four hours later, mice were mock infected with PBS or orally infected with 1 × 10^7^ CFU strain DBS770. The experiments were performed twice with a total of 15 mice per group.

### Infection with C. rodentium with the *tna* operon.

C. rodentium with *tna* operon was generated by inserting the *tna* operon from EHEC in the *lacZ* locus of the WT C. rodentium (DBS770) strain. Microbiota-depleted mice were infected with 1 × 10^7^ CFU of either WT DBS770 or DBS770 with the *tna* operon. On the indicated days, feces were collected for colonization, qRT-PCR, and survival analysis. The experiments were performed twice with a total of 15 mice per group.

### Colony enumeration.

The feces from infected mice were collected and placed in microcentrifuge tubes on ice. Feces were resuspended in PBS, normalized to feces weight, and plated on plates with the appropriate media and antibiotics. The colonies were counted, and the statistical comparison between groups were performed by unpaired Mann-Whitney *U* test.

### Tissue collection for mass spectrometry, RNA isolation, and qRT-PCR.

At the indicated time points, mice were euthanized, and the colon tissue and content were collected. The tissue was washed in PBS twice to remove any residual fecal content. The content and tissues were snap-frozen in liquid nitrogen. Mass spectrometry analysis was performed to estimate the amount of indole and tryptophan in those fractions. The feces from infected mice were collected in tubes. The tubes were snap-frozen in liquid nitrogen and stored in −80°C until use. RNA was isolated from individual mouse fecal pellets using RNeasy Power Microbiome kit (Qiagen) per the manufacturer’s instructions. Monocolonization of *B. theta* exacerbates C. rodentium infectivity (30), causing mice to be very sick with very little fecal content obtained from each mouse. In such cases, fecal samples from three or four mice in the same cage were combined and RNA was extracted from the combined material. A total of 10 mice per group were used to obtain RNA from the experiment. qRT-PCR was performed as described earlier using Quantstudio 6 flex (Applied Biosystems). *rpoA* was used as an internal control for Citrobacter rodentium. Significance was determined either by unpaired *t* test or Mann-Whitney *U* test as stated in figure legends.

### Mass spectrometry analysis of tryptophan and indole.

Tissue and content/feces weights were homogenized in a threefold volume of methanol (3 × weight of tissue in grams = volume methanol in milliliters; total homogenate volume [in milliliters] = 4 × weight of tissue). Homogenates were made using a BeadBug microtube homogenizer run for 2 min at 2,800 rpm and BeadBug-prefilled tubes with 3.0-mm zirconium beads (catalog no. Z763802; Sigma). Standards were made by spiking 100 μl of control BM homogenate with various known concentrations of indole and processed like samples. Tryptophan standards used BM homogenate diluted 1:5,000 in methanol and were spiked with various known concentrations of tryptophan. The tissue homogenate samples were incubated at room temperature for 10 min. Samples were spun at 13,000 for 5 min. The supernatant (100 μl) was combined 50:50 with ddH_2_O containing 0.2% formic acid and 100 ng/ml 13Trp IS and spun again, The supernatant was transferred to an HPLC vial w/insert and analyzed by HPLC/MS. The Qtrap 6500 and analytical conditions follow: buffer A was ddH_2_O plus 0.1% formic acid; buffer B was MeOH plus 0.1% formic acid; column, Agilent C_18_ XDB column with 5-μm packing and 50- by 4.6-mm size. The gradient conditions were as follows: 0 to 1.0 min, 0% buffer B; 1 to 1.5 min, gradient to 100% buffer B; 1.5 to 3.5 min, 100% buffer B; 3.5 to 4.0 min, gradient to 0% buffer B; 4 to 5 min, 0% buffer B. The ion source/gas parameters follow: CUR = 30, CAD = 9, IS = 5500, TEM = 200, GS1 = 20, GS2 = 10. Indole transition, 118.0 to 91.0; tryptophan transition, 205.09 to 188; 13 Trp IS transition: 216.043 to 155.2. Samples were injected twice, once at 20 μl with the indole standard curve and again at 5 μl with the tryptophan standard curve. This was done instead of making dilutions.

### Quantification and statistical analysis.

For all *in vitro* experiments (qRT-PCR and FAS) and mass spectrometry data, a Student’s unpaired *t* test was used when comparing two groups and a one-way ANOVA was used when comparing more than two groups. Multiple-comparison correction using Bonferroni-Dunn method was used wherever required. For experiments with mice, the nonparametric Mann-Whitney *U* test was used to determine the statistical significance. Statistics for survival analysis was calculated using log rank (Mantel-Cox) test. A *P* value of <0.05 was considered significant. Detailed information on the numbers of biological samples and animals used can be found in the figure legends.

### Data and software availability.

RNA-Seq data can be accessed using accession number (GSE119440) at the NCBI GEO database.
