# Erratum for Kumar et al., “Indole Signaling at the Host-Microbiota-Pathogen Interface”

**DOI:** 10.1128/mBio.03318-19

**Published:** 2020-02-04

**Authors:** Aman Kumar, Vanessa Sperandio

**Affiliations:** a Department of Microbiology, University of Texas Southwestern Medical Center, Dallas, Texas, USA; b Department of Biochemistry, University of Texas Southwestern Medical Center, Dallas, Texas, USA

## ERRATUM

Volume 10, no. 3, e01031-19, 2019, https://doi.org/10.1128/mBio.01031-19. The wild-type (WT) control group represented in Fig. 5D for the expression of *cpxR* was inadvertently duplicated from WT controls of *espA* from Fig. 5C. Figure 5D is corrected below. The changes do not affect any of the results of the study. The corrected figure version of Fig. 5 is shown here.

**FIG 5:**
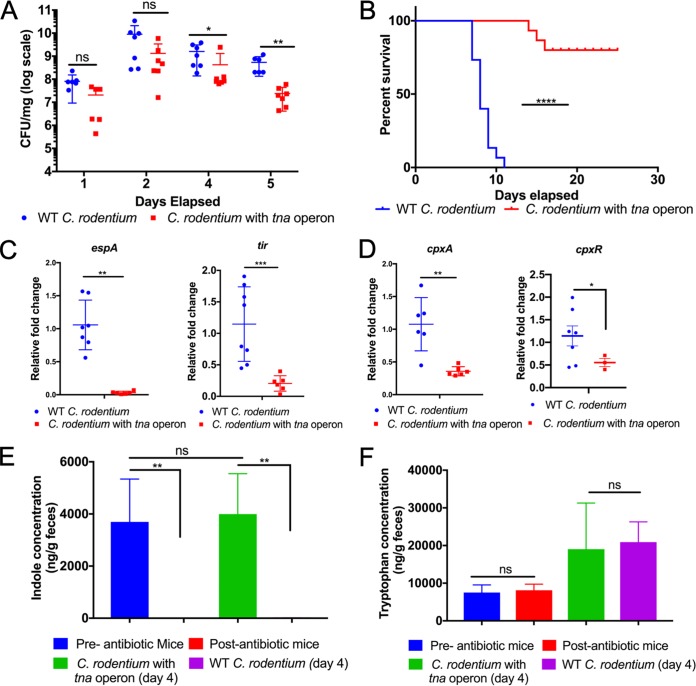
Indole limits C. rodentium colonization and pathogenesis in mice.

