# Supplementary material for: Indole Signaling at the Host-Microbiota-Pathogen Interface
Source: mBio. 2019 Jun 4;10(3):e01031-19. doi: 10.1128/mBio.01031-19 (PMC6550529; doi:10.1128/mBio.01031-19)
Supplement: FIG S1 [file mBio.01031-19-sf001.pdf]

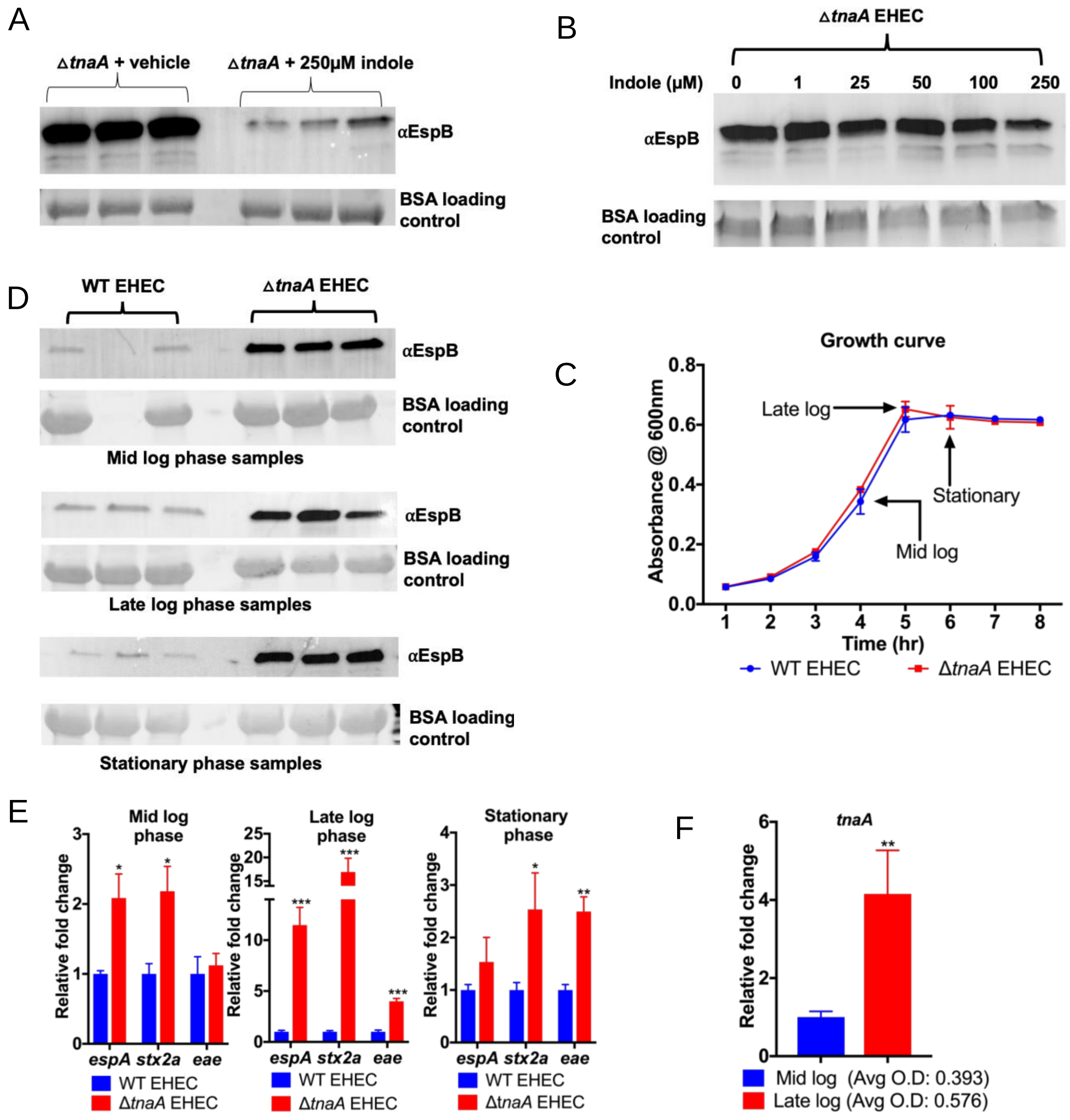


**FIG S1** High expression of virulence genes is observed in late logarithmic phase. (A and B) Δ*tnaA* EHEC was grown anaerobically. Cells were harvested in late log phase and supernatant were probed with EspB antibody. Bacterial cells were grown with (A) 250μM indole, or (B) a gradient of indole ranging from 1-250μM. BSA was used as a loading control. (C) Growth curve of WT and Δ*tnaA* EHEC grown anaerobically. Arrow heads indicate growth phases namely mid-logarithmic (mid log), late-logarithmic (late log) or stationary phase samples. (D) Western blot

analysis on the secreted protein EspB obtained from WT and ∆*tnaA* EHEC samples grown anaerobically in mid log, late log, and stationary phase. BSA was used as a loading control. (E) qRT-PCR analysis of select virulence genes from WT and ∆*tnaA* EHEC samples grown anaerobically and harvested in either mid log, late log, or stationary phase. Statistical significance was calculated using unpaired t-test followed by multiple comparison by Bonferroni-Dunn method. (F) Expression of *tnaA* obtained from WT and ∆*tnaA* EHEC samples grown anaerobically until mid-log or late log phase. *P*-value was determined by unpaired t-test. . Error bars indicate standard deviation (SD). Subjects with asterisks (*), (**), and (***) indicate $p<0.05$, $p<0.01$, and $p<0.001$ respectively.
