# Supplementary material for: Indole Signaling at the Host-Microbiota-Pathogen Interface
Source: mBio. 2019 Jun 4;10(3):e01031-19. doi: 10.1128/mBio.01031-19 (PMC6550529; doi:10.1128/mBio.01031-19)
Supplement: FIG S2 [file mBio.01031-19-sf002.pdf]

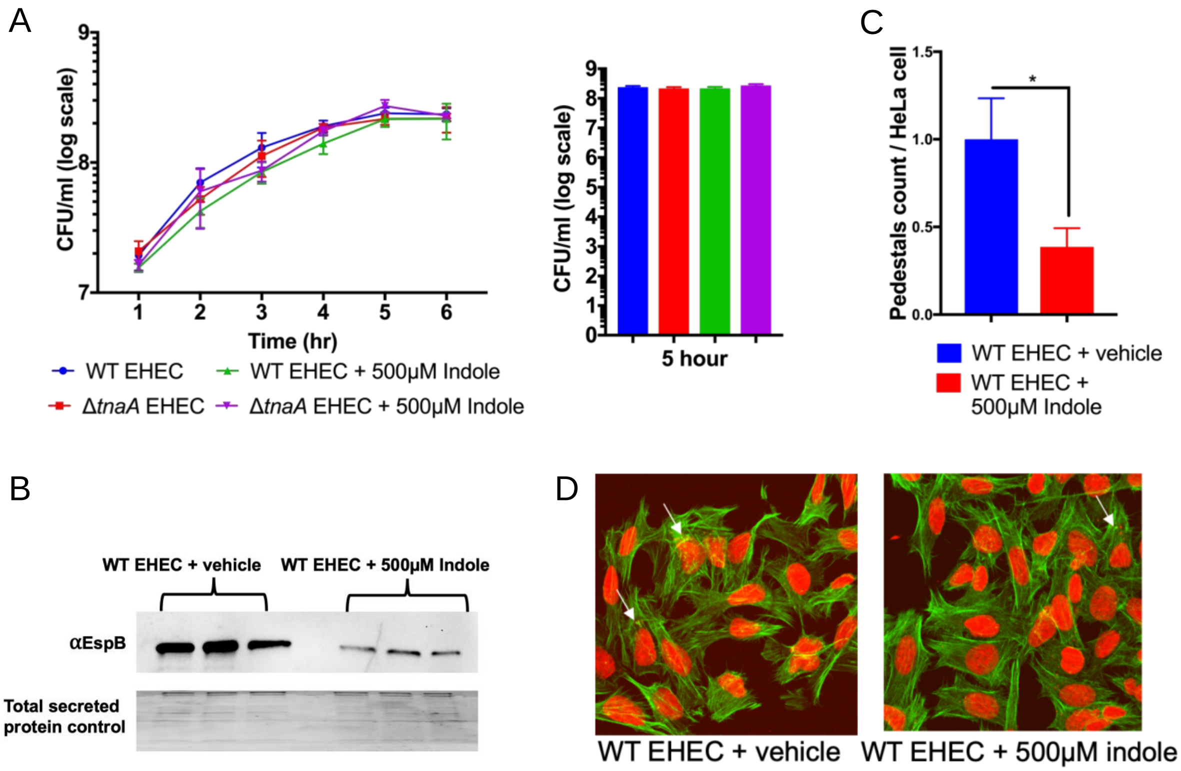

FIG S2. High indole concentration decreases EHEC virulence without affecting its growth. (A) Growth curves of WT, Δ*tnaA* EHEC with or without indole showing number of viable cells at different time points. Experiments were performed anaerobically, and a 5-hour time point was used to perform experiments representing late logarithmic (log) phase. Error bars indicate standard deviation (SD). (B) WT EHEC was grown anaerobically in the presence or absence of 500μM indole. Bacterial cells were harvested in late log phase (5 hour) and supernatant were probed with EspB antibody. All data are representative of three independent experiments. (C) Fluorescein actin staining (FAS) of HeLa cells infected with WT EHEC in the presence or absence of 500μM indole. The number of pedestals per HeLa cell were quantified and statistical significance were determined using unpaired t-test, * represents $p<0.05$.

(D) Representative image of FAS imaging for HeLa cells infected with WT EHEC in the presence or absence of indole. Pedestals were visualized as green puncta (white arrow heads). All experiments were performed at least twice with three biological replicates.
