# Supplementary material for: Indole Signaling at the Host-Microbiota-Pathogen Interface
Source: mBio. 2019 Jun 4;10(3):e01031-19. doi: 10.1128/mBio.01031-19 (PMC6550529; doi:10.1128/mBio.01031-19)
Supplement: FIG S3 [file mBio.01031-19-sf003.pdf]

A

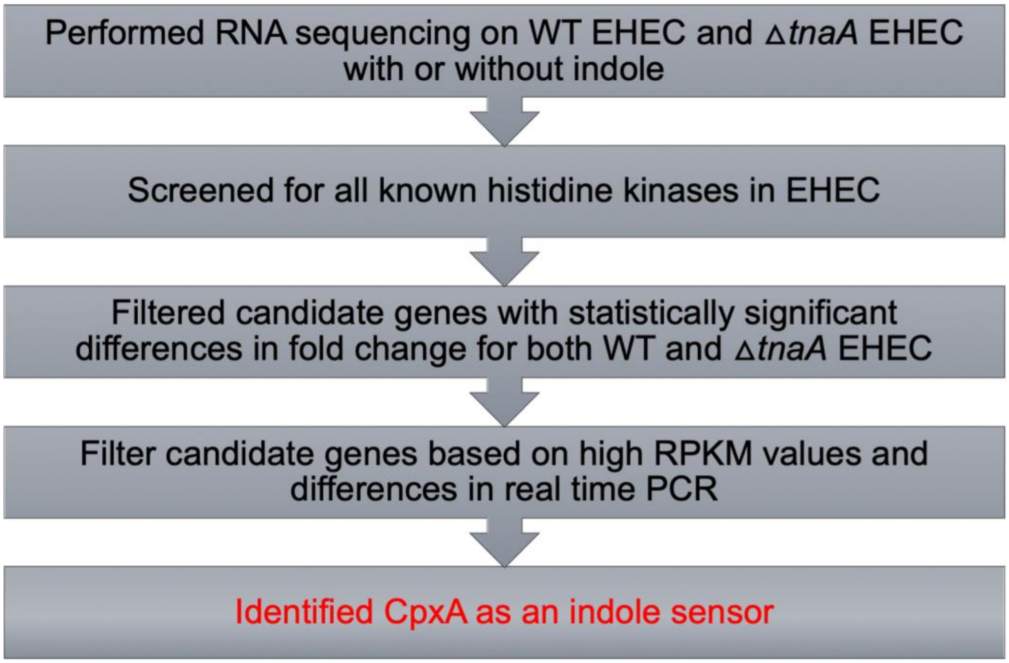

B

	Linear total RKPM				Fold Change		p-value	
	**TI**	**T**	**WI**	**W**	**WI vs W**	**TI vs T**	**WI vs W**	**TI vs T**
uhpB	1.43	2.15	2.66	2.64	1.01	0.67	0.976	0.285
narX	18.92	21.37	28.26	27.56	1.03	0.89	0.865	0.558
basS	7.79	11.99	13.55	15.75	0.86	0.65	0.709	0.423
envZ	14.61	14.97	19.49	23.19	0.84	0.98	0.484	0.939
baeS	10.98	13.89	13.26	14.55	0.91	0.79	0.453	0.075
rscC	355.36	538.14	522.42	446.80	1.17	0.66	0.407	0.301
arcB	210.80	274.98	322.44	272.59	1.18	0.77	0.388	0.041
dcuS	12.72	27.11	43.80	70.46	0.62	0.47	0.175	0.231
yehU	21.95	35.72	42.67	52.66	0.81	0.61	0.165	0.056
cheA	0.91	1.28	1.47	2.38	0.62	0.71	0.162	0.200
glnG	20.51	31.58	21.81	29.61	0.74	0.65	0.141	0.030
evgS	11.39	32.59	32.08	47.87	0.67	0.35	0.115	0.137
kdpD	42.72	18.25	25.89	17.78	1.46	2.34	0.098	0.020
cusA	4.93	4.37	3.14	5.62	0.56	1.13	0.095	0.425
narQ	11.88	17.25	15.66	29.86	0.52	0.69	0.071	0.461
phoP	87.96	64.70	145.94	82.29	1.77	1.36	0.067	0.057
phoQ	20.14	35.63	33.70	54.90	0.61	0.57	0.053	0.145
torS	3.10	5.52	5.05	11.26	0.45	0.56	0.036	0.113
creC	5.43	10.03	9.50	12.62	0.75	0.54	0.033	0.040
yojN	15.93	43.87	22.63	38.63	0.59	0.36	0.029	0.012
rstB	20.90	29.71	18.78	39.13	0.48	0.70	0.028	0.395
barA	6.75	20.96	14.98	31.61	0.47	0.32	0.023	0.035
cpxA	29.30	63.59	58.24	109.42	0.53	0.46	0.019	0.021
hydH	65.16	56.27	100.41	44.37	2.26	1.16	0.004	0.545

FIG S3. CpxA identified as an indole sensor. (A) Flowchart showing the method used to screen for candidate indole sensor. (B) List of all known histidine kinases of EHEC. RNA sequencing

experiment was performed anaerobically with WT and Δ*tnaA* EHEC in the presence or absence of indole. Based on reads per kilobase million (RKPM) values and statistical analysis, *cpxA* was identified as a candidate gene differentially regulated in the presence of indole. RNA seq results were analysed in ArrayStar. Statistical significance was calculated using Student's t-test followed by FDR (Benjamini Hochberg) correction. A p-value of less than 0.05 was considered significant.
