# Supplementary material for: Indole Signaling at the Host-Microbiota-Pathogen Interface
Source: mBio. 2019 Jun 4;10(3):e01031-19. doi: 10.1128/mBio.01031-19 (PMC6550529; doi:10.1128/mBio.01031-19)
Supplement: FIG S4 [file mBio.01031-19-sf004.pdf]

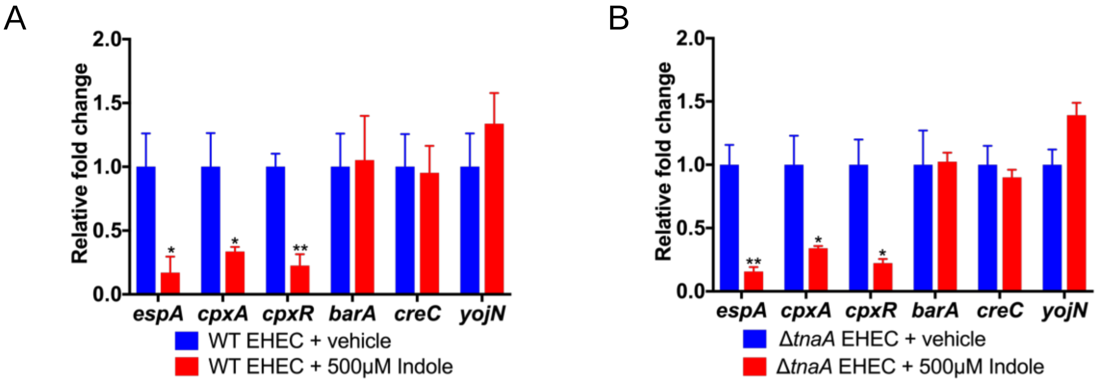

C

	Linear Total RKPM				Fold Change		p-value	
**Name**	**TI**	**T**	**WI**	**W**	**WI vs W**	**TI vs T**	**WI vs W**	**TI vs T**
rssB	97.54	93.15	99.84	93.02	1.07	1.05	0.65	0.9
phoB	14.84	18.02	20.68	19.46	1.06	0.82	0.86	0.46
acrB	65.69	183.5	175.41	217.8	0.81	0.36	0.26	0.02
arcA	9.63	76.74	22.91	131.06	0.17	0.13	0.01	0.002
fimZ	0.44	0.32	0.63	0.2	3.14	1.38	0.43	0.86
cusR	5.9	3.59	4.59	2.61	1.76	1.64	0.28	0.2
kdpE	13.81	8.32	12.26	4.16	2.95	1.66	0.01	0.29
torR	68.63	60.16	72.29	54.01	1.34	1.14	0.02	0.55
phoQ	20.14	35.63	33.7	54.9	0.61	0.57	0.05	0.15
phoP	87.96	64.7	145.94	82.29	1.77	1.36	0.07	0.06
narL	64.59	83.1	108.89	138.58	0.79	0.78	0.06	0.24
rstA	2.11	3.37	4.4	6.72	0.65	0.63	0.29	0.36
cheY	9.65	14.02	18.35	17.94	1.02	0.69	0.92	0.32
cheB	0.91	1.28	1.47	2.38	0.62	0.71	0.16	0.2
uvrY	68.55	70.46	76.91	100.59	0.76	0.97	0.29	0.94
baeR	47.73	33.11	55.85	32.91	1.7	1.44	0.06	0.11
yehT	29.05	43.72	61.81	46.61	1.33	0.66	0.1	0.18
narP	6.13	9.35	10.69	14.67	0.73	0.66	0.17	0.4
rcsB	355.36	538.14	522.42	446.8	1.17	0.66	0.41	0.3
evgA	41.15	37.8	64.98	26.56	2.45	1.09	0.18	0.94
ompR	143.79	121.61	188.81	156.74	1.2	1.18	0.17	0.42
uhpA	0.77	1.1	0.43	1.85	0.23	0.7	0.14	0.3
glnL	16.87	14.24	19.57	15.66	1.25	1.19	0.14	0.48
cpxR	90.67	169.15	152.92	249.51	0.61	0.54	0.05	0.02
hydG	12.71	12.33	18.21	12.17	1.5	1.03	0.21	0.9
basR	43.13	32.75	74.74	29.54	2.53	1.32	0.09	0.34
dcuR	20.74	25.66	29.01	36.51	0.79	0.81	0.06	0.64
creB	5.09	11.62	10.88	17.83	0.61	0.44	0.11	0.01

FIG S4. Indole signaling occurs through CpxA-CpxR node. (A and B) qRT-PCR analysis for select statistically significant differentially regulated histidine kinases (*cpxA, barA, creC,* and *yojN*) and response regulator (*cpxR*) obtained from (A) WT and (B) Δ*tnaA* EHEC grown anaerobically in the presence or absence of 500μM indole. Expression for *espA* was also included as a control. Fold change was calculated relative to endogenous control, *rpoA.*

Statistical analysis was performed using unpaired t-test followed by multiple comparison by Bonferroni-Dunn method. Subjects with asterisks (*), (**) indicate $p<0.05$, $p<0.01$.

(B) List of all known response regulators of EHEC. Based on reads per kilobase million (RKPM) values and statistical analysis, *cpxR* (cognate response regulator of *cpxA*) was noted to be similarly differentially regulated in the presence of indole. RNA seq results were analysed in ArrayStar. Statistical significance was calculated using Student's t-test followed by FDR (Benjamini Hochberg) correction. A p-value of less than 0.05 was considered significant.
