# Supplementary material for: Indole Signaling at the Host-Microbiota-Pathogen Interface
Source: mBio. 2019 Jun 4;10(3):e01031-19. doi: 10.1128/mBio.01031-19 (PMC6550529; doi:10.1128/mBio.01031-19)
Supplement: FIG S5 [file mBio.01031-19-sf005.pdf]

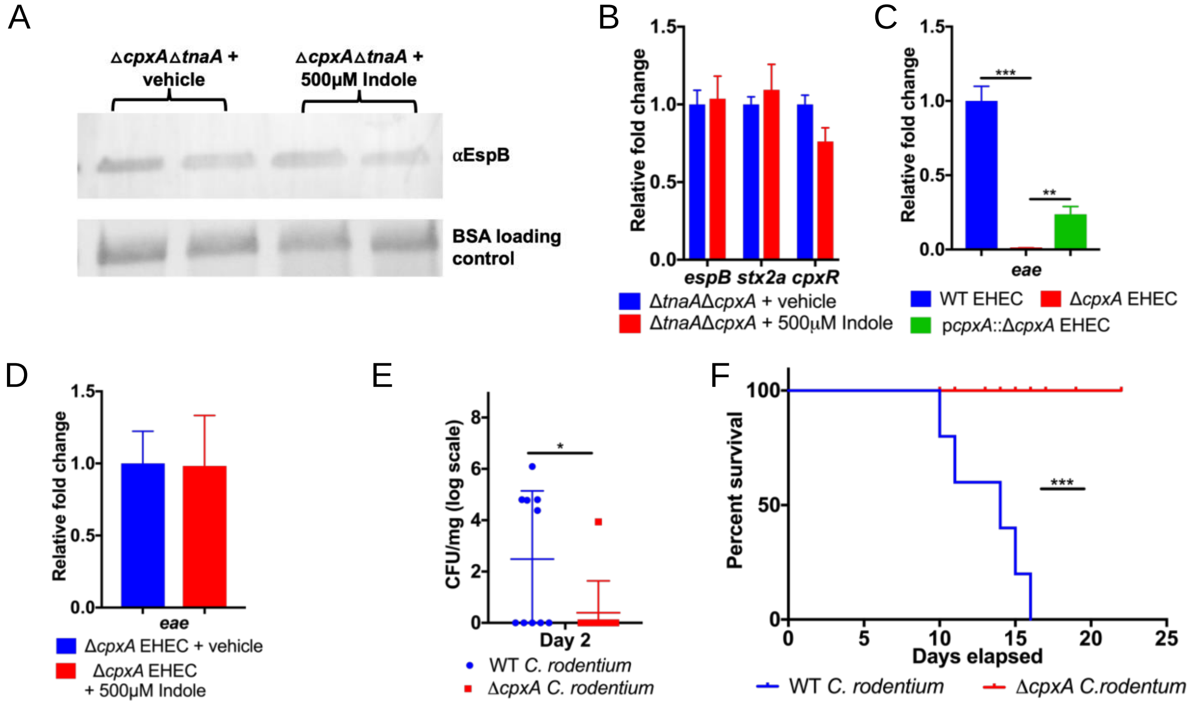

FIG S5 A *cpxA* mutant is indole irresponsive and is attenuated for pathogenesis. (A and B) *cpxA* deficient EHEC strains were grown anaerobically in the presence or absence of indole. (A) Western blot analysis on the secreted protein EspB, (B) qRT-PCR analysis on the expression of select genes. These experiments were performed in Δ*tnaA* EHEC background strain to avoid the effects of endogenous indole. (C) qRT-PCR analysis on representative virulence gene (*eae*) indicating Δ*cpxA* EHEC is attenuated for virulence gene expression. WT, Δ*cpxA* and p*cpxA* (complemented strain) EHEC strains were grown anaerobically. *P* values were determined by using one-way ANOVA. (D) qRT-PCR expression analysis of Δ*cpxA* EHEC strains with and without indole. All qRT-PCR experiments are representative of at least two independent experiments. Error bars indicate standard deviation (SD). Subjects with asterisks (**), (***) represent p<0.01, and p<0.001. (E) Mice were infected with either WT *C. rodentium* or Δ*cpxA* *C. rodentium*. Fecal samples were collected day 2 post-infection to assess the colonization levels

of these strains in mice. Each data point represents an individual mouse. *P*-value was determined by using Mann-Whitney *U*-test. Error bars indicate standard error of mean (SEM), * indicate $p<0.05$. (F) Survival analysis of mice infected with either WT *C. rodentium* or △*cpxA C. rodentium.* A total of n=10 mice per group were used for the study. Statistical significance was calculated using log rank (Mantel-Cox) test, *** indicates $p<0.001$.
