# Supplementary material for: Indole Signaling at the Host-Microbiota-Pathogen Interface
Source: mBio. 2019 Jun 4;10(3):e01031-19. doi: 10.1128/mBio.01031-19 (PMC6550529; doi:10.1128/mBio.01031-19)
Supplement: FIG S6 [file mBio.01031-19-sf006.pdf]

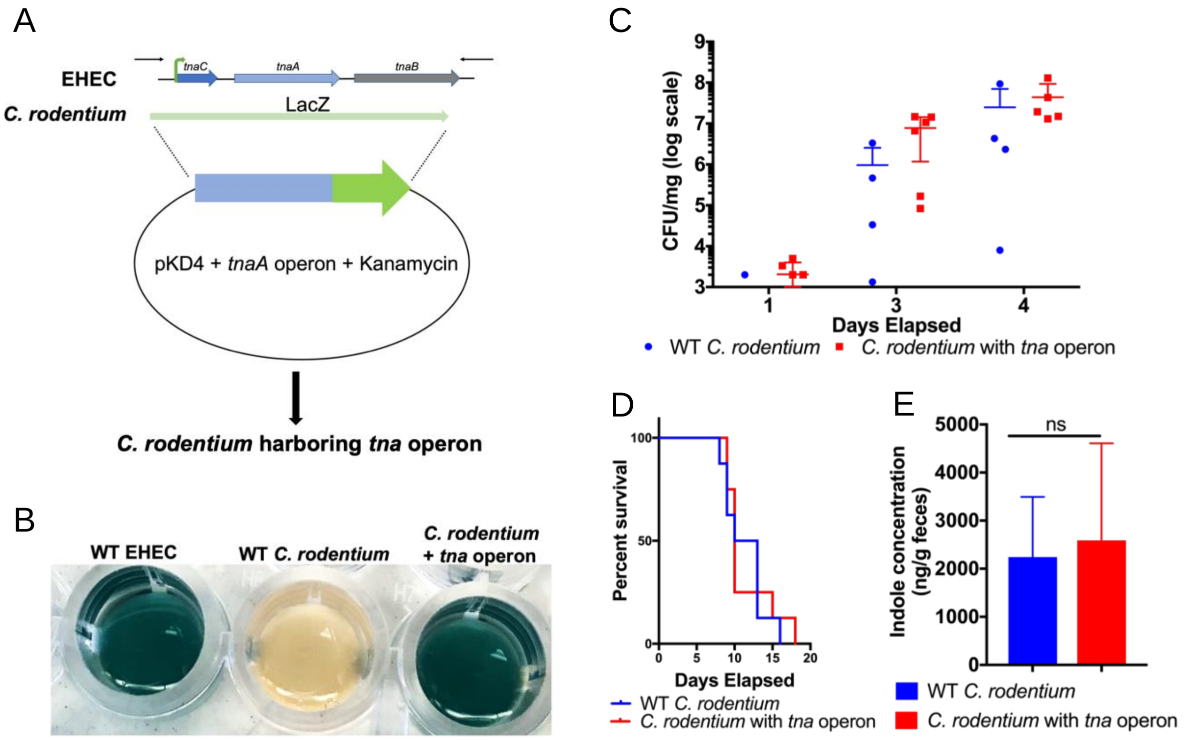

FIG S6 Self-produced indole or microbiota-derived indole dictates *C. rodentium* infectivity in a similar fashion. (A) Cartoon depicting the method used to engineer the *tna* operon from EHEC into the *lacZ* locus of *C. rodentium* (*C. rodentium* naturally lacks *tna* operon and cannot produce indole). (B) Colorimetric assay testing the designed *C. rodentium* ability to produce indole using DMACA reagent. (C) Conventional C3H/HeJ mice were infected with either WT *C. rodentium* or *C. rodentium* with *tna* operon strain. Fecal samples were collected on days 1, 3 and 4 post infection to assess for bacterial colonization. Each data point indicates an individual mouse. Error bars indicate standard error of mean (SEM). (D) Survival analysis of mice infected with either WT *C. rodentium* or indole producing *C. rodentium.* N = 10 mice per group were used for the experiment. (E) Mass spectrometry measurements of indole obtained from fecal samples collected on day 4 from mice infected with either WT *C. rodentium* or engineered *C. rodentium.* N= 4 mice per group, ns is not significant.
