# Supplementary material for: Indole Signaling at the Host-Microbiota-Pathogen Interface
Source: mBio. 2019 Jun 4;10(3):e01031-19. doi: 10.1128/mBio.01031-19 (PMC6550529; doi:10.1128/mBio.01031-19)
Supplement: FIG S7 [file mBio.01031-19-sf007.pdf]

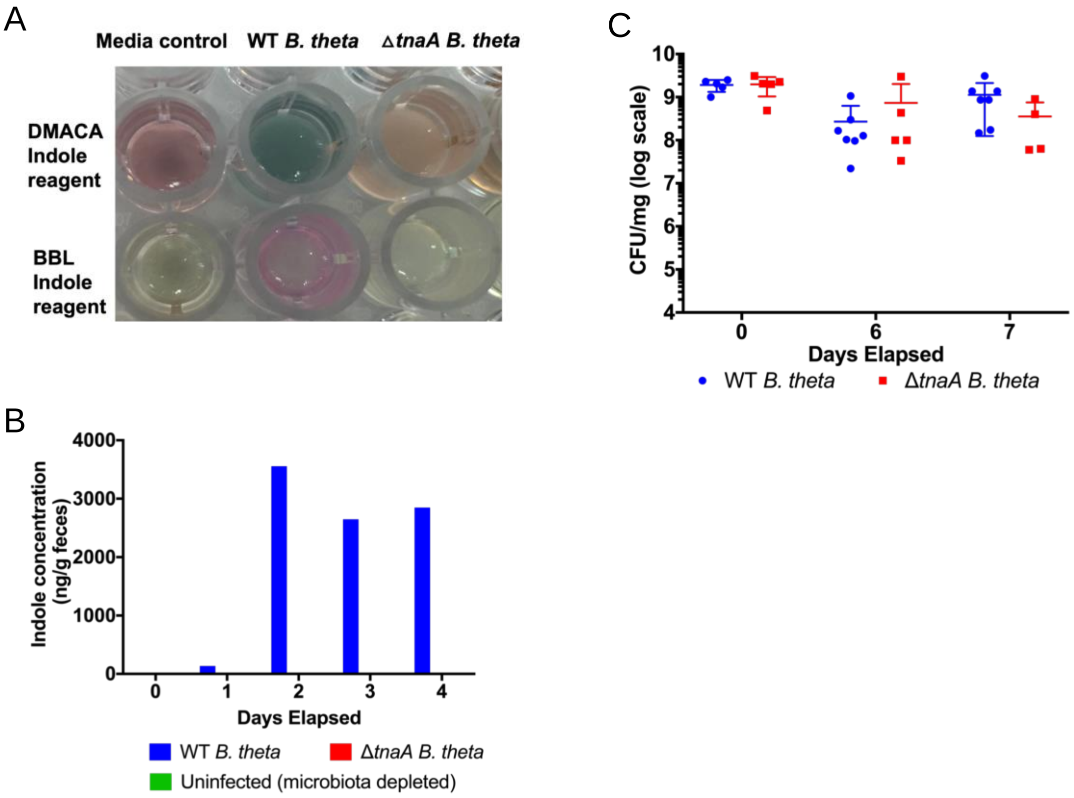

FIG S7 *B. theta* murine colonization is not affected by *tnaA*. (A) Colorimetric assay to confirm △*tnaA B. theta* inability to produce indole using two different reagents. (B) Time course of indole measurement from microbiota depleted mice left uninfected, or mono-colonized with either WT or △*tnaA B. theta* using mass spectrometry. N =4 mice were used per group. Samples from each group were pooled together to estimate the indole concentration.

(C) Colonization profile of *B. theta* during *C. rodentium* murine infection. Each data point represents an individual mouse.
