# Supplementary material for: Indole Signaling at the Host-Microbiota-Pathogen Interface
Source: mBio. 2019 Jun 4;10(3):e01031-19. doi: 10.1128/mBio.01031-19 (PMC6550529; doi:10.1128/mBio.01031-19)
Supplement: TABLE S1 [file mBio.01031-19-st001.pdf]

Table S1. Bacterial Strains.

**Bacterial Strains**		
**Strain**	**Description**	**Reference**
	EHEC	
WT EHEC	*E. coli* O157:H7 86-24 Clinical isolate	(Griffin et al., 1988)
Δ*tnaA*	isogenic *tnaA* deletion mutant	This study
Δ*cpxA*	isogenic *cpxA* deletion mutant	This study
Δ*tnaAcpxA*	isogenic *cpxAtnaA* double deletion mutant	This study
	Bacteroidetes thetaiotaomicron (B. theta)	
VPI-5482	Wild-type, GentR	ATCC 29148
Δ*tnaA*	isogenic *tnaA* deletion mutant	This study
	Citrobacter rodentium	
DBS770	*C. rodentium*	(Mallick et al., 2012)
AK-DBS770-*tna*	*C. rodentium* with *tna* operon	This study
Δ*cpxA*	isogenic *cpxA* deletion mutant	This study
**Plasmids**		
pKD4	λ red template plasmid	(Datsenko and Wanner, 2000)
pKD46	λ red helper plasmid	(Datsenko and Wanner, 2000)
pCP20	λ red helper plasmid	(Datsenko and Wanner, 2000)
pACYC177	Cloning vector	New England Biolabs
pET21a	Expression vector	Millipore
