# Supplementary material for: Indole Signaling at the Host-Microbiota-Pathogen Interface
Source: mBio. 2019 Jun 4;10(3):e01031-19. doi: 10.1128/mBio.01031-19 (PMC6550529; doi:10.1128/mBio.01031-19)
Supplement: TABLE S2 [file mBio.01031-19-st002.pdf]

Table S2.Oligonucleotides used in this study.

Primer	Sequence 5’ to 3’
rpoA F	GTGACCCTTGAGCCTTTAGAG
rpoA R	ACACCATCAATCTCAACCTCG
espA F	AGCTATTTGAGGAACTCGGTG
espA R	CATCTTTTGTGCCGTGGTTG
espB F	GGTCAAGGCTACGGAAAGTG
espB R	TCTTCAGCAAAGTCAGAGGC
tir F	GAGGGAGTCAAATAGCGGTG
tir R	ATCTGAACGAAGGCTGGAAG
eae F	TGGGATGTTCAACGGTAAGTC
eae R	TTTAACCTCAGCCCCATCAC
stx2a F	TGTCTGAAACTGCTCCTGTG
stx2a R	GATATTCTCCCCACTCTGACAC
rpoZ F	AGGACGCTGTAGAGAAAATTGG
rpoZ R	GCGCGATTACAGTGGTTTTATC
Ier F	CGAGAGCAGGAAGTTCAAAGTG
ler R	ACACCTTTCGATGAGTTCCG
escC F	CTGAAGACAATGGCAAGTAATGG
escC R	ACTGCATTAAGACGTGGATCAG
escV F	GAGTGCAAAAGGAAAGCCAG
escV R	ATGATACCAGCAATAGCGTCC
cpxA F	TGGTGAAAGCAAGGAACTGG
cpxA R	TGATTGGCTTTGATGGTCTCG
cpxR F	GATGACTATCTCCCGAAACCG
cpxR R	ATCAACTTCCAGTGTCGGTG
tnaA F	AGGGATTAGAACGCGGTATTG
tnaA R	CGGAGTTACTGGTGATGGTTG
barA F	GCTGTTTTGTATCGGTATTGCG

barA R	AGCATAAATCCTTCCACTCGG
creC F	GCGAATTTTATGGGCCAGC
creC R	AGGAACGGGCTTATTGTCAG
yojN F	TTTGGCTATACGGAAGGGTTC
yojN R	TTCCAGCACCTTATCAACCG
arcA F	ATGGTTGGGAACTGGACATC
arcA R	CAGCACGGGACTGAATTTTG
CR_espA F	ACGAGGTAACAACCATGCGAGTGT
CR_espA R	CTGCCTGGCATTGCTTTCCAGAAT
CR_tir F	ATCAGATATCTCGCAAGCTCG
CR_tir R	CAACTCCATCTCCCATTCCTG
CR_eae F	TGCGAAAGATACAGCCCTTAG
CR_eae R	ACCTCTGCCGTTCCATAATG
CR_rpoA F	ACGTCAGCCGGAAGTGAAAGAAGA
CR_rpoA R	AGCGGACAGTCAATTCCAGATCGT
CR_cpxA F	TCCGTAACTTCATTGGTCAGG
CR_cpxA R	CGGTCGAATCAGGTAAAGCTG
CR_cpxR F	TGAACATCTCAGCCAGGAAG
CR_cpxR R	CACGCAGAGTTTTAAACCACG
CR_escV F	GGGCGATGAAGTTTGTAAAAGG
CR_escV R	CCAACCGACAATACAGAAAACAG
LR_CR_*tnaA_LacI*_F	CTGCTGCACCTCGCCCGCCAGGTTGCGCAGCTCTCACCA AAGATGTGAACGCATAACGTGATTCGATTCACATTTAAA CAATTTC
AK_prom_*tnaA*_F_EHEC	GTGATTCGATTCACATTTAAACAATTTC
AK_Kan_pkd4_SOE_F	CGTGTTACCTAAATTTGGCTAAAGCAAGCGAACCGGAAT TGC
AK_*tnaB*_R	TTAGCCAAATTTAGGTAACACG
AK_*lacZ*_*prsM*_kn_R	GCCAGTACGGCGGCCAGTGAATCAGTATTGAGGTTCATG GTTCAGTCCTCAGAAGAACTCGTCAAGAAGGCG
AK_pkd4_Kan_R	TCAGAAGAACTCGTCAAGAAGGCG
AK_tnaA_BT_P1_F	GCCACTAGTTGCATGGGTAACAAATACGACTTATGA

AK_tnaA_BT_P2_R	CCATCTTGATTTTCCATGATTCAGCA
AK_tnaA_BT_P3_F	TGCTGAATCATGGAAAATCAAGATGGACCGTTCAGTTAGAACGATTGTC
AK_tnaA_BT_P4_R	GCCACTAGTTGAAGCATCTTTGCATATTGATAAACGG
